# Louping Ill in Goats, Spain, 2011

**DOI:** 10.3201/eid1806.120220

**Published:** 2012-06

**Authors:** Ana Balseiro, Luis J. Royo, Claudia Pérez Martínez, Isabel G. Fernández de Mera, Úrsula Höfle, Laura Polledo, Nelson Marreros, Rosa Casais, Juan F. García Marín

**Affiliations:** Servicio Regional de Investigación y Desarrollo Agroalimentario, Gijón, Asturias, Spain (A. Balseiro, L.J. Royo, R. Casais);; Universidad de León, León, Spain (C. Pérez Martínez, L. Polledo, J.F. García Marín);; Instituto de Investigación en Recursos Cinegéticos, Ciudad Real, Spain (I.G. Fernández de Mera, Ú. Höfle, N. Marreros)

**Keywords:** louping ill, goats, Spain, Asturian strain, viruses, Flavivirus, louping ill virus, vector-borne infections

## Abstract

Although louping ill affects mainly sheep, a 2011 outbreak in northern Spain occurred among goats. Histopathologic lesions and molecular genetics identified a new strain of louping ill virus, 94% identical to the strain from Britain. Surveillance is needed to minimize risk to domestic and wildlife species and humans.

Louping ill is a zoonotic disease caused by a neurotropic, single-stranded, 40 to 50–nm RNA virus, which has been classified in the family *Flaviviridae*, genus *Flavivirus*. Louping ill virus belongs to a subgroup antigenically related to viruses known as the tick-borne encephalitis viruses (TBEVs) of Europe (*1*). These viruses are medically considered to be the most common flaviviruses in Europe and Asia (*2*). Among them, the TBEVs, which infect thousands of humans per year (*3*), are related to louping ill virus. Most reports of louping ill virus originate from the British Islands; knowledge with regard to the strain from Spain is limited. Because it is a tick-transmitted disease, the distribution of louping ill is closely associated with the distribution of the primary vector, the tick *Ixodes ricinus*. It mainly affects sheep and red grouse (*Lagopus lagopus scotica*), but many other species have been reported to be susceptible, including dogs, llamas, alpacas, goats, pigs, and humans (*2*).

Infection with a strain of louping ill virus from Spain was first reported for sheep in the Basque region of northern Spain in 1987. For several years during the spring, mortality rates for lambs and yearlings in infected flocks were high (*4*). The causative agent was identified as Spanish sheep encephalitis virus (SSEV) (*5*). Additional studies failed to isolate the virus from ticks (*6*) in the region where the first cases occurred, indicating that prevalence of SSEV, if present, was low. To our knowledge, no cases of encephalitis caused by a flavivirus in ungulates in Spain have been described since then.

Also to our knowledge, no cases of tick-borne flavivirus infection in humans have been reported in Spain, although 1 case of tick-borne encephalitis in a person from southwestern France was considered to have been caused by an SSEV subtype virus (*3**,**7*). Therefore, except for the 1 person in France, SSEV infections seem to be restricted to sheep and to the Basque region of northern Spain. We report suspected infection of a herd of Bermeya goats (an endangered breed of Asturian goats) with a TBEV.

## The Cases

In September 2011, a herd of 70 adult goats was purchased in southern Asturias and then moved to northern Asturias. Within 1 month, 1 goat became ill. The first sign was hindleg lameness, which quickly progressed to incoordination, fever, tremors, and bulging eyes. The goat died 2 days later, after which 17 other goats (including 2 goatlings born on the farm) showed the same signs and died over a 4-month period. Many ticks were found on these animals (≈10–15 ticks/goat), and Butox (Merck, Madrid, Spain) was applied.

Necropsy was performed on 2 adult goats; gross lesions were recorded and special attention was paid to the nervous system. Samples for histopathologic examination were taken from the brain (cerebrum, midbrain, cerebellum, and brain stem), spinal cord, liver, kidney, adrenal glands, lungs, spleen, and gastrointestinal tract. They were fixed in 10% neutral-buffered formalin, and 4-µm hematoxylin and eosin–stained sections were produced.

Samples of brain tissue were also taken for molecular analysis. Total RNA was extracted (TRIzol reagent; Gibco BRL, Grand Island, NY, USA), treated with DNase 1 (Takara Bio Inc., Kyoto, Japan), and reverse transcribed into single-stranded cDNA by using random hexamers (First Strand cDNA Synthesis Kit for reverse transcription PCR (RT-PCR) [avian myeloblastosis virus]); Roche Diagnostics, Indianapolis, IN, USA). Real-time RT-PCR primers designed to detect all viruses in the family *Flaviviridae* (*8*) were used in a conventional RT-PCR protocol, and a 231-bp amplicon was detected and sequenced (BigDye Terminator, version 3.1, Cycle Sequencing Kit protocol; Applied Biosystems, Foster City, CA, USA). To compare the phylogenetic relationships of the isolate virus with other representative TBEV strains, we constructed an unweighted pair-group method analysis tree in MEGA 5 (*9*), by using published TBEV sequences (*8*) and the Three Arch Rock Island strain of Tyuleniy virus as an outgroup.

**Figure 1:**
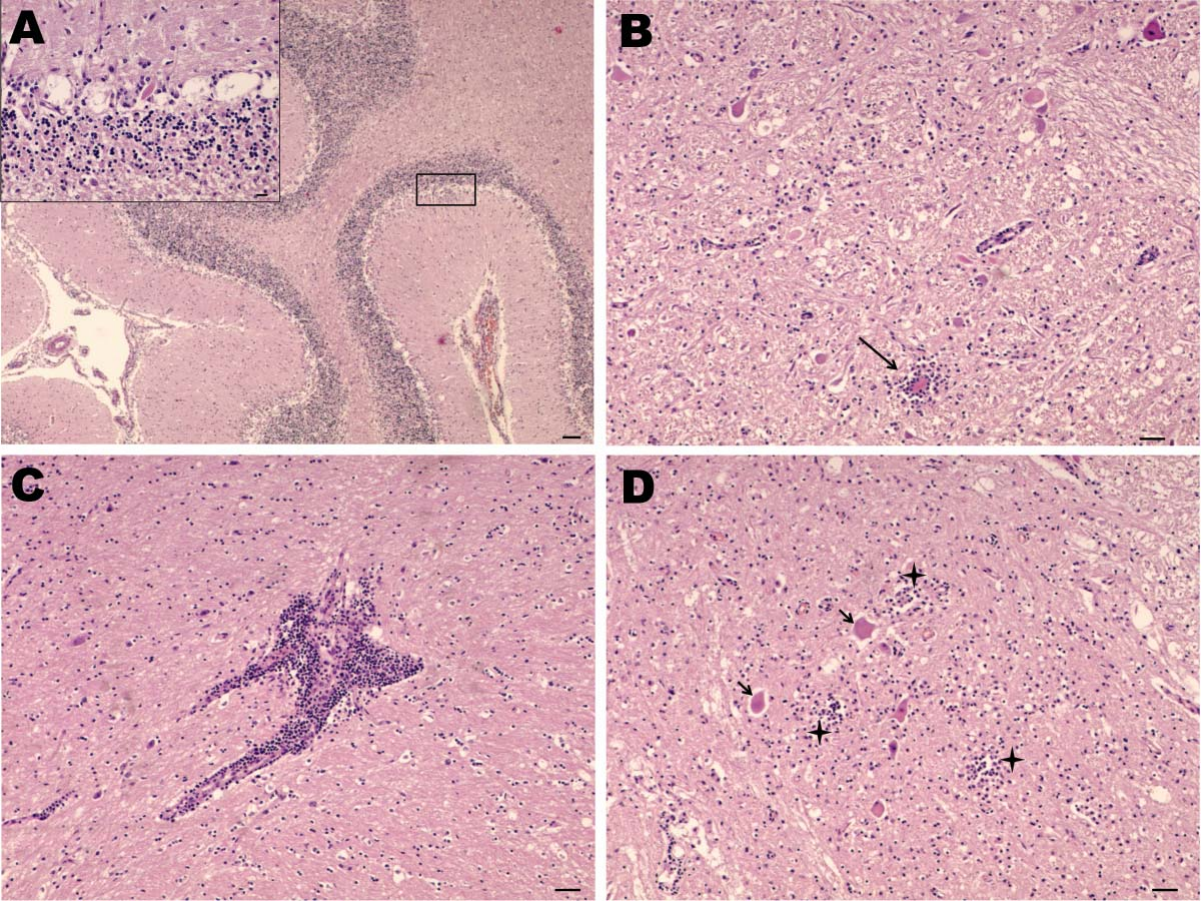
Nonsuppurative encephalitis in goat affected by louping ill. A) Cerebellum with necrosis of Purkinje cells. Hematoxylin and eosin (H&E) stain; scale bar = 100 µm. Inset: necrosis of Purkinje cells. H&E stain; scale bar = 20 µm. B) Midbrain. Area of neurophagia (arrow) surrounded by microglial cells. Necrosis of neurons can be also seen. H&E stain; scale bar = 50 µm. C) Lymphoid perivascular cuff in midbrain. H&E stain; scale bar = 50 µm. D) Spinal cord, gray matter. Focal microgliosis (crosses) and neurons undergoing necrosis (arrows). H&E stain; scale bar = 50 µm.

During necropsy of the 2 goats, no gross lesions were found. No histopathologic lesions were found in any organ system except the central nervous system (Figure 1). The cerebellum showed necrosis of Purkinje cells and neurons. Histopathologic examination of the rest of the brain revealed a mild meningeal infiltration with widespread lymphocytic perivascular cuffs and evidence of neurophagia and gliosis, characterized by degenerating neurons surrounded by glia cells. These lesions were concentrated in the hypothalamus and midbrain and were more severe in the medulla oblongata and spinal cord. The histopathologic lesions observed in these goats were indistinguishable from those caused by louping ill virus (*10*).

**Figure 2:**
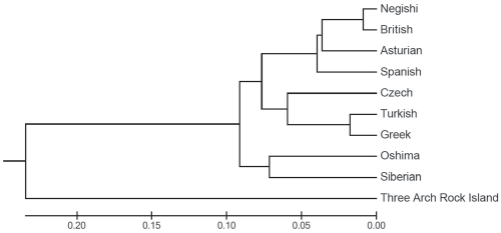
Phylogenetic relationships of the Asturian strain louping ill virus with representative tick-born encephalitis viruses. Phylogenetic and molecular evolutionary analyses of the virus were conducted using MEGA version 5 (*9*). Scale bar indicates branch length, proportional to the number of nucleotide substitutions. The Three Arch Rock Island virus was included as an outgroup.

Molecular genetic studies enabled identification of the virus. The sequence (Genbank accession no. JQ646028) was 94% identical to the strain from Britain (EU074000) and 93% identical to the strains from Spain (EU074016) and the Negishi virus (EU074002), thus confirming identity of the Asturian strain louping ill virus. Phylogenetic analysis, conducted by using the unweighted pair-group method analysis tree (Figure 2) shows how the new virus strain is related to the strains from Britain and Spain.

## Conclusions

Histopathologic lesions together with molecular genetic results enabled a definitive diagnosis: tick-borne encephalitis caused by an Asturian strain of louping ill virus. These cases confirm the infection in species other than sheep in Spain and the presence of the virus in areas of northern Spain other than the Basque region. An epidemiologic survey confirmed that no clinical signs were observed for the source herd in southern Asturias. However, a few years ago, another herd, located in the same area to which the affected herd described here had been moved, showed similar signs; 8 of 20 goats died. Although a diagnosis was not confirmed for this earlier outbreak, the facts suggest that the virus might have been in this area for several years.

The concern over finding louping ill in this area lies in its zoonotic potential. Numerous cases of human infection have been described (*11*). Humans can become infected in a variety of ways. Infections have been naturally acquired in persons who had direct contact with infected animals, for example on the farm or in laboratory settings (*11**,**12*). The virus can also be transmitted by direct mucous or respiratory pathways. Another route for infection is the consumption of milk from infected goats or products (cheese, butter, or yogurt) made from milk from infected goats (*13*). The presence of the virus in milk could represent a public health hazard if the milk is not pasteurized.

The Bermeya goat is considered to be at high risk for extinction, and many efforts have been made by breeders and the local administration of agriculture to limit the loss of genetic variability in these goats. The cases reported here provide an example of how an infectious disease can also reduce the local genetic resources. These cases, together with the case described in the Basque region, underline the need for a specific surveillance plan in northern Spain that focuses on ticks, wildlife species, and livestock. This plan will be crucial for determining the actual effects of louping ill on hunting, animal breeding, and human health.
